# Transcriptomic analysis revealed ferroptosis in ducklings with splenic necrosis induced by NDRV infection

**DOI:** 10.1186/s13567-025-01479-y

**Published:** 2025-03-09

**Authors:** Hongzhi Wang, Chenchen Jiang, Boyi Xu, Di Lei, Rendong Fang, Yi Tang

**Affiliations:** 1 College of Veterinary Medicine, Southwest University, Chongqing, China; 2 Institute of Animal Sciences of Chinese Academy of Agricultural Sciences, Beijing, China

**Keywords:** Novel duck orthoreovirus, splenic necrosis, macrophage, transcriptome, ferroptosis

## Abstract

Infection with novel duck reovirus (NDRV) results in severe splenic necrosis, leading to immunosuppression, secondary infections with other pathogens, and impairment of the immune effect of the vaccine. However, little is known about NDRV-induced spleen injury and its antagonistic mechanism on the host immune response. In this study, we conducted pathological and comparative transcriptomic analyses of NDRV-infected duck spleens. Our findings elucidated the histopathological progression of splenic necrotic foci formation following NDRV infection and identified splenic macrophages as the primary target cells. RNA-Seq analysis revealed differentially expressed genes that were enriched predominantly in immune system processes, signalling molecules and interactions, and pathways related to cell growth and necrosis. Notably, we observed a significant upregulation of ferroptosis during NDRV infection, characterized by the induction of specific metabolism-related genes such as TfR1, Hmox1, and STEAP3, alongside the downregulation of Fpn expression. Our findings collectively indicate the involvement of ferroptosis in spleen injury induced by NDRV infection. Investigating the mechanism of NDRV-induced ferroptosis in spleen macrophages will contribute to a comprehensive understanding of the pathogenesis associated with NDRV.

**Supplementary Information:**

The online version contains supplementary material available at 10.1186/s13567-025-01479-y.

## Introduction

Avian orthoreovirus (ARV) is a globally circulating immunosuppressive pathogen in poultry that infects various avian species, including chickens, ducks, geese, turkeys, and wild birds. It causes a variety of diseases, such as viral arthritis, tenosynovitis, growth retardation, and chronic respiratory diseases [1, 2]. ARVs are segmented RNA viruses whose genomes are divided into three groups (large, middle, and small) comprising 10 segments. The S1 gene encodes three proteins, resulting in 12 translation products in total [3]. Among these proteins, the σC protein plays a crucial role as the secondary capsid protein by facilitating virus attachment to host cells and inducing specific neutralizing antibody production during viral pathogenesis [4]. The σC protein-coding gene is also the most susceptible gene segment in ARVs under continuous immune selection pressure [5]. Furthermore, duplication of the ARV genome occurs after segment classification and packaging, which contributes significantly to its high susceptibility to gene recombination during mixed infections [6]. In recent years, the accumulation of point mutations, genetic recombination, cross-host transmission, and successful adaptation of new variants of ARV have resulted in large-scale epidemics that pose significant challenges to poultry health and economic production [1, 7, 8].

China is a prominent country for waterfowl breeding. Since the initial report of ARV infection in Muscovy ducks in 1997, a series of diseases have emerged, including “Muscovy duck mosaic liver disease” caused by Muscovy duck orthoreovirus infection, “Gosling hemorrhagic necrotic hepatitis” caused by goose orthoreovirus infection [9], “Duck necrotizing hepatitis” caused by novel Muscovy duck orthoreovirus infection [10], and “Duck splenic necrosis disease” caused by duck orthoreovirus infection [11]. In 2017, we reported the prevalence of a novel variant of duck orthoreovirus (NDRV), characterized by increased pathogenicity, increased clinical mortality rates, and severe splenic necrosis in infected ducks [8]. However, the pathogenesis of NDRV-induced splenic necrosis and severe immunosuppression remains elusive. Elucidating the target cells invaded by NDRV in the spleen and unravelling the molecular mechanism through which NDRV regulates host cell injury are imperative.

The investigation of ARV-induced host cell damage was initiated in 1989 [12]. ARV can trigger the apoptosis of multiple avian and mammalian cell types [13] while also inducing autophagy through the PI3K/Akt/mTOR pathway and interacting with the host candidate target protein EF1α1 [14, 15]. During ARV infection, autophagy is induced in Vero and DF-1 cells during the early to middle stages of infection, followed by apoptosis induction during the middle and late stages [16]. In terms of immunosuppression, O’Hara proposed that ARV exhibits tropism for macrophages [17], and the target cells of Muscovy DRV infection also include macrophages and lymphocytes in the spleen. However, despite ARV being an important immunosuppressive pathogen, relatively limited attention has been given to understanding the mechanism of immune cell damage caused by ARV infection.

In conclusion, further investigations are needed to elucidate the mechanism underlying the development of splenic necrosis induced by NDRV and the molecular processes involved in host cell injury within the spleen. This study focused on ducklings infected with NDRV as an experimental model and employed haematoxylin‒eosin (HE) staining, immunohistochemistry, and RNA-Seq techniques to investigate both the dynamic changes and the molecular mechanisms associated with splenic necrosis. The identification of differentially expressed genes (DEGs) and novel forms of programmed cell death provides valuable insights for understanding the pathogenesis of NDRV.

## Materials and methods

### NDRV infection experiment in ducklings and sample collection

The 1-day-old healthy ducklings used in this study were obtained from a commercial hatchery located in Chongqing. After NDRV virus infection and negative serum antibody testing, each duckling in the infection group was intravenously injected with a dose of 10^5^ TCID_50_ of the NDRV strain (*n* = 45). As a control, 45 ducklings were similarly injected with saline. The ducklings were housed in groups within an isolator, and daily observations and symptom recording were conducted. At 1, 2, 3, 5, 7, 9, 11, 13 and 15 days post-infection (dpi), euthanasia was performed on the 6 ducklings (3 infected and 3 controls) via intravenous injection of pentobarbital sodium (New Asiatic Pharmaceutical, Shanghai China), followed by an examination of pathological changes in the spleen during autopsy. Concurrently collected spleen samples were prepared as paraffin-embedded tissue sections and single-cell suspensions. In accordance with conventional methods, histopathological observation was performed on paraffin sections stained with HE, and immunohistochemistry was employed for the detection of prepared paraffin sections [18].

### Repair of a single-cell suspension from the spleen

The spleens of ducklings in both the infected and control groups were promptly harvested on a bedstand treated with RNaseZap solution (Ambion, Austin, Texas, USA) and rinsed with precooled DEPC water to eliminate any traces of blood. The spleen was subsequently placed in a sterile dish, where the connective tissue and spleen capsule were carefully removed before being transferred to a 200-mesh cell sieve for mincing and grinding [19]. Following thorough grinding, the resulting cell suspension was collected by flushing the screen with whole blood and tissue cell mixture and then filtered through a 40-micron filter to obtain a duckled spleen cell suspension, which was utilized for total RNA extraction.

### RNA extraction, library preparation and RNA sequencing

The RNeasy Plus Micro Kit (Qiagen, Hilden, NRW, Germany) was utilized for the extraction of total RNA from suspensions following the manufacturer’s instructions. The quantity and quality of the RNA were assessed via an Agilent 2100 Bioanalyzer (Agilent, Palo Alto, CA, USA). RNase-free DNase I (Ambion, Austin, Texas, USA) treatment was subsequently applied to eliminate potential genomic DNA contamination in the RNA samples. Purification was performed via the use of poly-T oligo-attached magnetic beads (Ambion, Austin, Texas, USA), and fragmentation into 200–300 bp fragments occurred. First-strand cDNA synthesis was performed with reverse transcriptase with random 6-base primers utilizing RNA as a template. The second-strand cDNA was subsequently synthesized on the basis of the first-strand cDNA, and the 3' ends of the DNA fragments were adenylated. PCR amplification enriches library fragments, which are then selected on the basis of fragment size, resulting in a library of 300–400 bp in length [20]. Finally, paired-end sequencing with 150 bp reads was conducted in the library via an Illumina HiSeq 2000 sequencer at Personal Biotechnology Co., Ltd. (Personal Bio, Shanghai, China).

### Quality analysis, mapping, and transcriptome assembly

Cutadapt software was used for the removal of adaptors, poly-N sequences, and low-quality data. The quality assessment of the sequencing data was performed via FastQC software. The filtered clean reads were de novo spliced via Trinity software to obtain contigs for each sample, which were subsequently assembled as unigene sequences. The clean reads were aligned to the Anas platyrhynchos genome via TopHat2 software (Baltimore, MD, USA), and the mapping rate of the sequencing data was calculated [21].

### Differential expression analysis

The clean reads were normalized as transcripts per million (TPM) via the reads per kilobase million (RPKM) method, and differential gene expression between the NDRV-infected and mock groups was calculated. DESeq software was used to analyse the differential expression of genes [22]. Differences in mRNA expression were considered significant if the adjusted *P* value (FDR) was < 0.05 and the log_2_|fold change| was > 1.5.

### Annotation and enrichment analysis

TopGO software was used for Gene Ontology (GO) enrichment analysis, and the gene list and the number of each term were determined on the basis of DEGs annotated by GO terms. Following GO functional annotation, Kyoto Encyclopedia of Genes and Genomes (KEGG) automatic annotation was employed to identify the locations of the DEGs in signalling pathways and regulatory relationships [23]. Significantly enriched GO terms were identified with a corrected *P* < 0.05, whereas KEGG pathways with a *P* < 0.05 were considered significantly enriched by DEGs.

### Quantitative real‑time PCR validation

To validate the reliability of the RNA-seq data, a subset of 12 DEGs was randomly selected for quantitative real-time PCR (qRT‒PCR). Each sample was subjected to three biological replicates, and the duck β-action gene was utilized as an internal reference gene. The qRT‒PCRs were conducted on a LightCycler96 Real-Time PCR instrument (Roche, USA) with specific primers designed via Primer 5 software (Additional file 1). The relative expression levels were normalized to those of the reference gene β-actin via the 2^−ΔΔCt^ method. All the data are presented as the means ± standard deviations from three replicates. Statistical differences in gene expression were analysed via one-way analysis of variance in SPSS software (IBM, IL, USA). A *P* < 0.05 was considered statistically significant.

## Results

### Pathological alterations associated with splenic necrosis induced by NDRV

**Figure 1:**
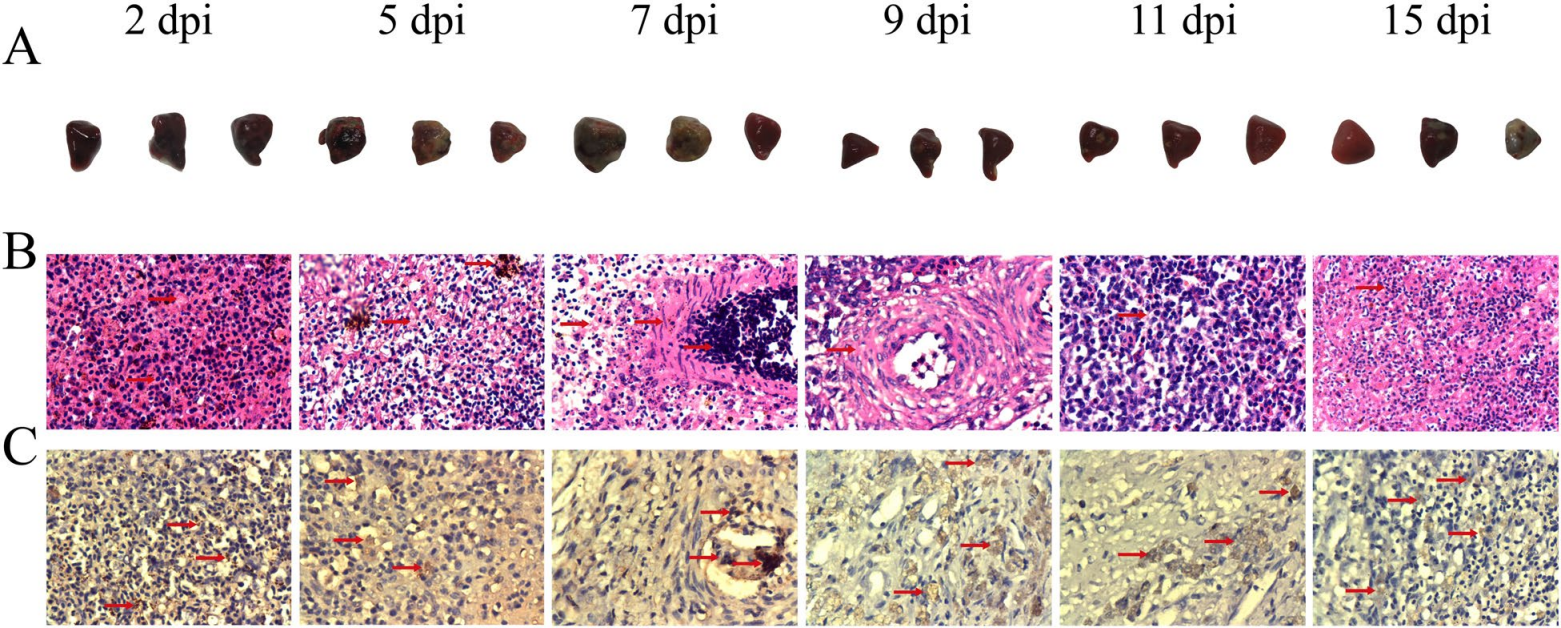
**Pathological alterations and immunohistochemical analysis of the spleen in ducklings infected with NDRV**. **A** Pathological alterations in the spleen of infected ducklings at 2, 5, 7, 9, 11 and 15 dpi; **B** Histopathological changes in the spleen stained with HE (400 ×); **C** Immunohistochemical analysis of these spleen paraffin sections (400 ×).

The pathological examination revealed splenomegaly and scattered necrotic lesions in the NDRV-infected duckling at 2 dpi, which became severe splenic necrosis at 5 and 7 dpi, with almost complete replacement of normal tissue by necrotic foci. However, gradual resolution of splenic necrosis was observed at 15 dpi (Figure 1A). Histopathological examination revealed severe splenic pulp congestion at 2 dpi. At 5 dpi, localized tissue necrosis and disintegration were observed, accompanied by splenic congestion and reduced lymphocyte counts. At 11 dpi, extensive necrosis and disintegration of the splenic tissue, thickening of the vascular walls, and accumulation of a significant number of lymphocytes on the medial side were evident. At 15 dpi, fibrous tissue replaced the previously necrotic area in the spleen, while scattered lymphocytes remained (Figure 1B). At 5 dpi, brownish-yellow macrophages were observed. At 9 and 11 dpi, a significant number of positively stained macrophages were detected (Figure 1C).

### Identification of DEGs and gene expression profiles associated with splenic necrosis

**Figure 2:**
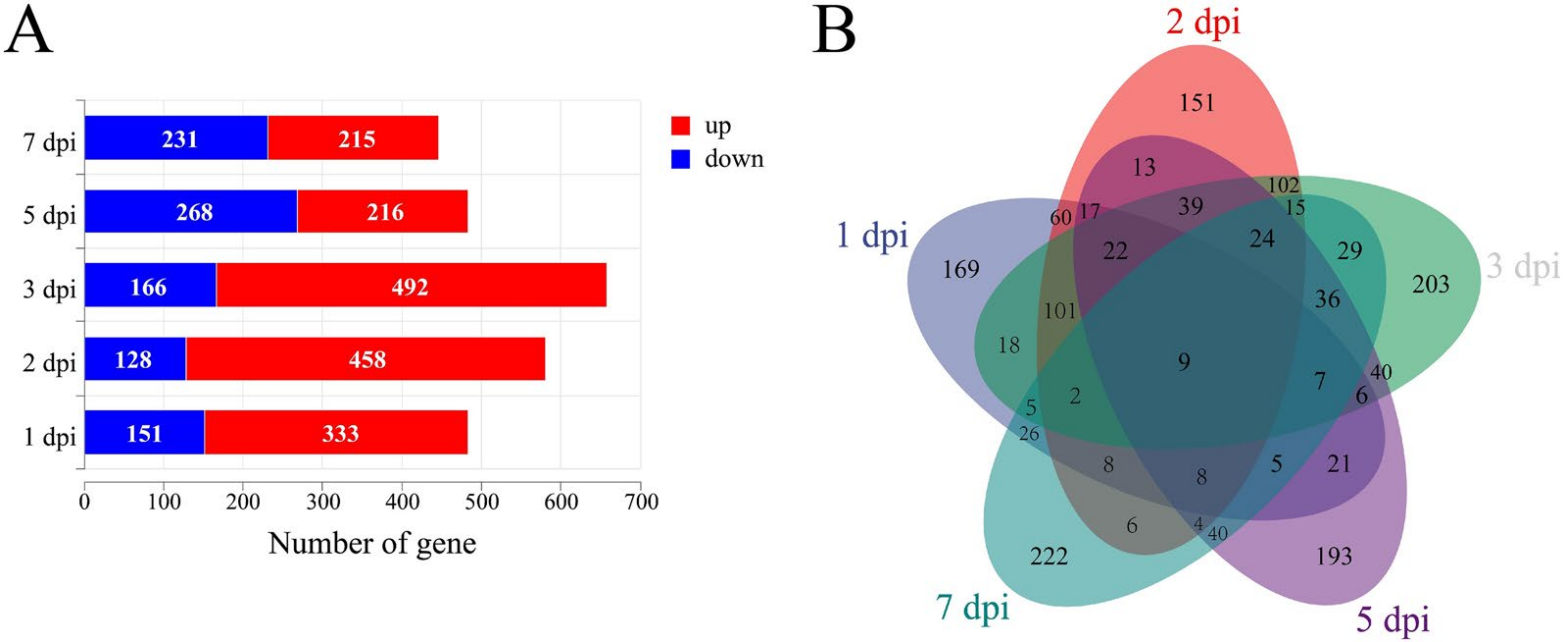
**DEGs were identified during splenic necrosis following NDRV infection**. **A** Statistics of the number of upregulated and downregulated DEGs; **B** Venn diagram of DEGs at different time points post infection.

The quality control results of the RNA-Seq data revealed that the average number of clean reads in the sequencing samples was 47 235 889, and the Q30 score ranged from 93.95 to 94.65% (Additional file 2). The comparison efficiency of the filtered clean reads to the reference genome was 88.10% for the control group and 88.56% for the infection group on average (Additional file 3). The DEGs were screened on the basis of the per-established criteria, resulting in the identification of a total of 2658 DEGs following NDRV infection, with 1714 genes exhibiting upregulation and 944 genes showing downregulation. The number of DEGs at 1 dpi was 446, whereas at 2, 3, 5, and 7 dpi, it reached staggering counts of 484, 658, 586 and 484, respectively (Figure 2A). A Venn diagram illustrates the overlap between these sets of DEGs at various time points following infection (Figure 2B).

### Analysis of GO terms and KEGG pathways enriched by DEGs

**Figure 3:**
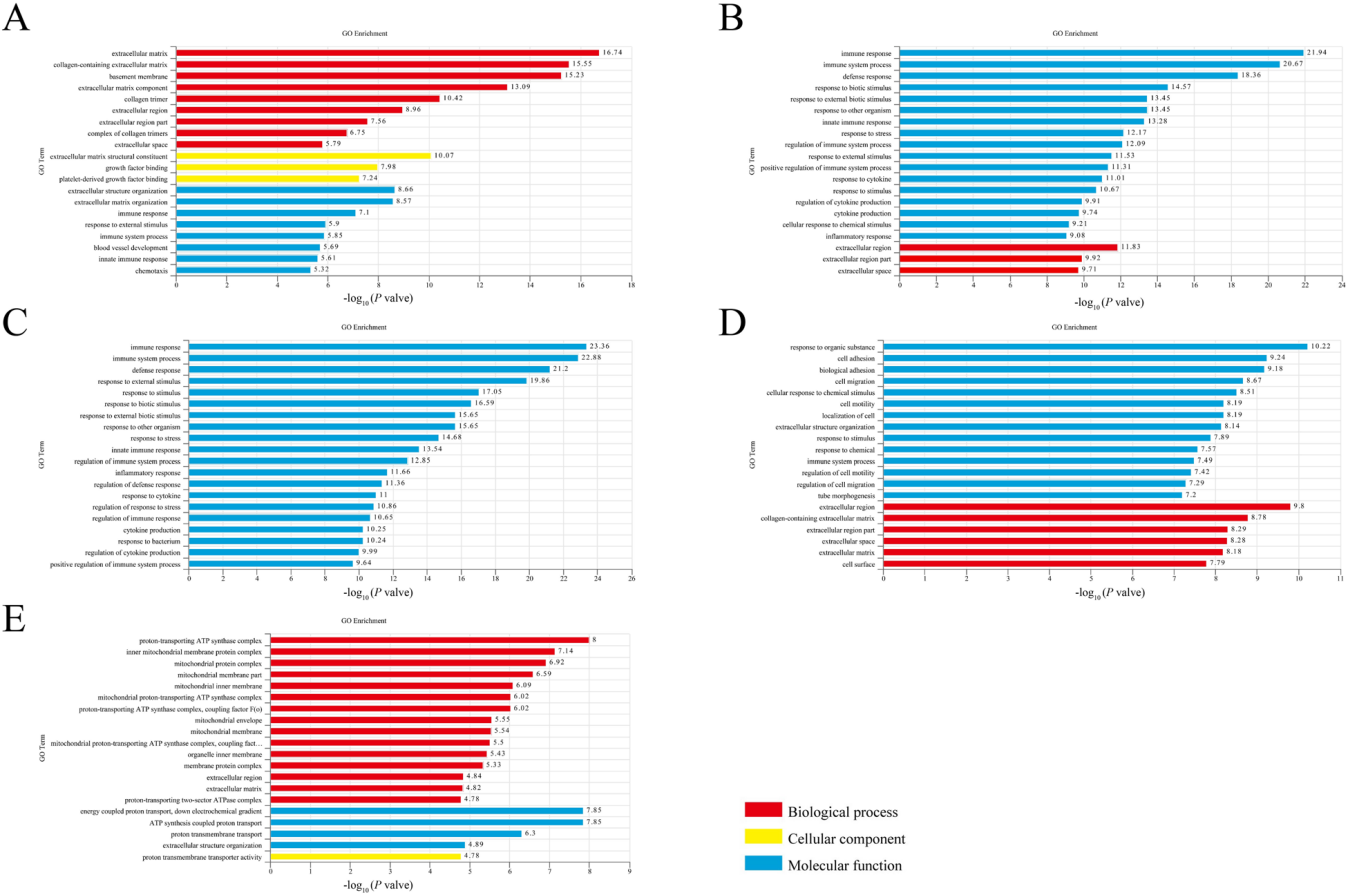
**Gene Ontology enrichment of DEGs**. **A** 1 dpi; **B** 2 dpi; **C** 3 dpi; **D** 5 dpi; **E** 7 dpi.

To elucidate the molecular mechanism underlying spleen necrosis and immunosuppression triggered by NDRV infection, we performed GO and KEGG enrichment analyses of the DEGs. The top 20 terms with highly significant differences at each time point after NDRV infection were associated primarily with biological functions such as the immune response, stress response, and biological regulation (Figure 3). Upon correlating the DEGs with systemic functions, a total of 14, 27, 22, 28, and 18 signalling pathways were significantly enriched at 1, 2, 3, 5, and 7 dpi, respectively (Additional file 4). Following NDRV infection in spleen cells, the biological functions of the DEGs were predominantly enriched in areas such as the immune system, signalling molecules and interactions, signal transduction, cell growth and death, and immune diseases.

### Meta-analysis of DEGs and enriched pathways implicated in cellular injury

**Figure 4:**
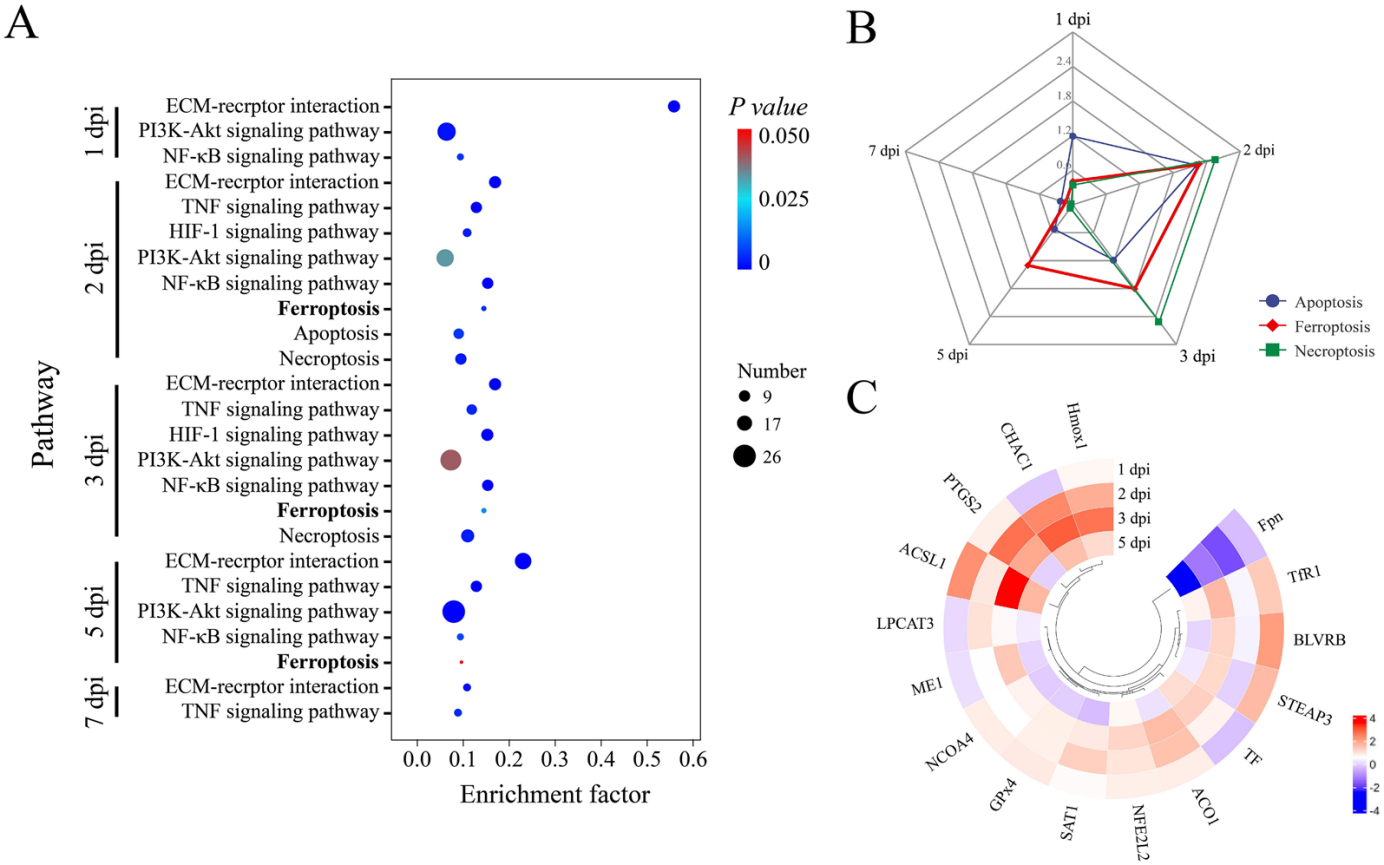
**Analysis of pathways and DEGs associated with cell death following NDRV infection**. **A** Enrichment of signalling pathways related to cell death after NDRV infection; **B** comparison of the significant differences in enriched modes of cell death; **C** expression of ferroptosis-related genes after NDRV infection.

To identify DEGs associated with cellular damage, we screened genes that were differentially expressed in relation to cell death. A total of 156 genes involved in the regulation of cell death were identified. Analysis of cell death patterns and signalling pathways revealed that apoptosis, necrosis, and ferroptosis were the primary modes of cell death observed in spleen cells following NDRV infection. The signalling pathways primarily involved in regulating cell death are the HIF-1 signalling pathway, the ECM‒receptor interaction pathway, the PI3K‒Akt signalling pathway and the TNF signalling pathway (Figure 4A). The significant differences observed among these three modes of cell death indicated the crucial role played by ferroptosis in inducing splenic necrosis during NDRV infection (Figure 4B).

### Expression profile of DEGs in the ferroptosis pathway

NDRV infection resulted in significant alterations in the expression of 16 genes associated with ferroptosis, with DEGs involved primarily in iron metabolism, the system *x*_*c*_^−^/GPx4 pathway and the lipid metabolism pathway. The expression of iron metabolism-related genes, including transferrin TF, TfR1, ACO1, and NCOA4, was upregulated following NDRV infection (Figure 4C). Conversely, the expression of Fpn was downregulated. The expression of Hmox1 was upregulated, thereby facilitating the release of iron ions from heme. The DEGs associated with iron metabolism suggest that NDRV may modulate host cell susceptibility to ferroptosis by altering intracellular iron homeostasis. The expression levels of GPx4 and the GSH metabolism-related genes BLVRB, ME1, and CHAC1 were found to be upregulated in various programs following NDRV infection. Additionally, the lipid metabolism-related genes ACSL1 and LPCAT3 presented increased expression. Furthermore, the regulation of ferroptosis by NDRV may involve the p53-SAT1-ALOX15 and ATG5-ATG7-NCOA4 pathways, as significant upregulation of the expression of the SAT1 and NCOA4 genes associated with this pathway was observed.

### Identification and functional analysis of DEGs related to innate immunity

**Figure 5:**
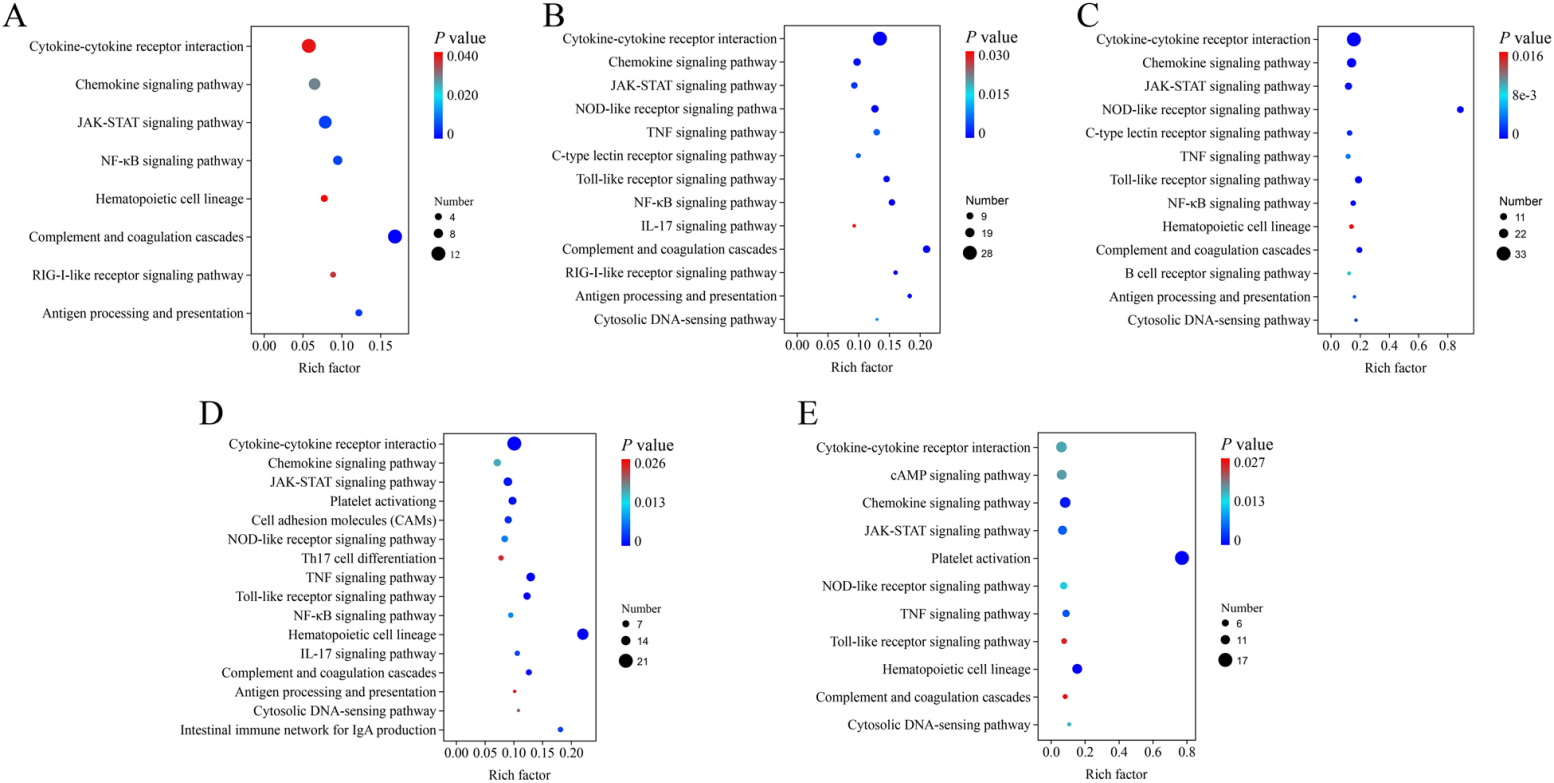
**The immune system pathways enriched during NDRV infection (P < 0.05)**. **A** 1 dpi; **B** 2 dpi; **C** 3 dpi; **D** 5 dpi; **E** 7 dpi.

Immunosuppression has emerged as a pivotal determinant of host injury induced by NDRV. The screening and functional identification of DEGs associated with innate immunity revealed that the biological functions related to the immune response were associated primarily with antigen processing and presentation, the RIG-I-like receptor signalling pathway, the Toll-like receptor signalling pathway, the JAK-STAT signalling pathway, the NF-κB signalling pathway, and cytokine‒cytokine receptor interactions following NDRV infection (Figure 5). Toll-like receptors are crucial for the recognition of viral infection by the host. The JAK-STAT and NF-κB signalling pathways play pivotal roles in the cellular inflammatory response, immune response, and signal transduction, underscoring the importance of the host’s innate immune response following NDRV infection. The expression of pattern recognition receptors (TLR2, TLR5, etc.), signalling transducers and regulators (STAT1, STAT2, IRF7, CARD9, etc.), chemokines (CCL3, CCL5, CCL19, etc.), antigen-presenting proteins (CD3, CD8), interleukins and their receptors (IL8, IL10, IL12A, etc.) was significantly upregulated following infection.

### Validation of the expression profile of DEGs

**Figure 6:**
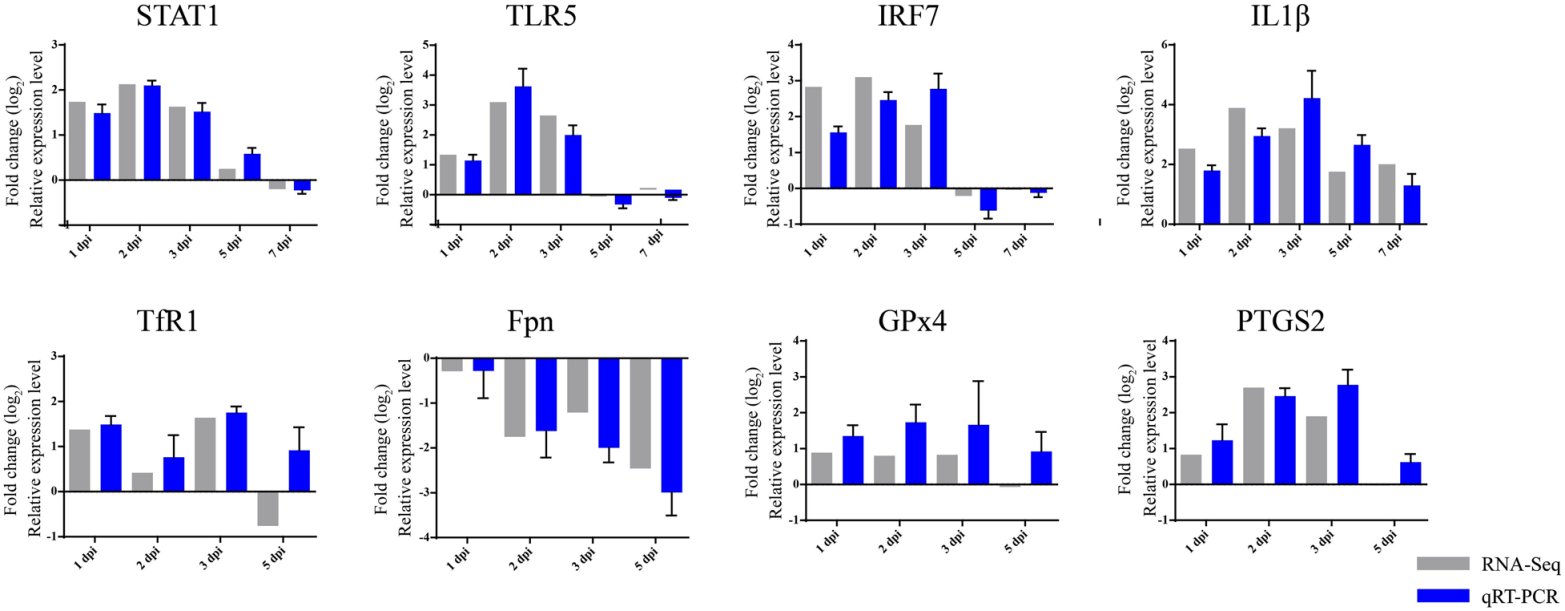
**qRT‒PCR was used to verify the levels of differentially expressed genes identified via RNA-Seq**. The expression was estimated via the 2^−ΔΔCT^ method, and the qRT‒PCR data are presented as the means ± standard deviations (*n* = 3).

To further validate the expression profile of the DEGs identified via RNA-Seq, we selected 12 DEGs associated with innate immunity and ferroptosis for qPCR validation. As shown in Figure 6, the changes in the expression of the DEGs detected via qRT‒PCR were consistent with the results obtained via RNA‒Seq analysis. However, there were instances where the expression trends at specific time points did not exhibit a complete correlation (TfR1). Overall, the qPCR validation substantiated the reliability of the RNA-Seq data.

## Discussion

NDRV infection induces duck splenic necrosis, which subsequently leads to immunosuppression, secondary infections by other pathogens, and impairment of vaccine-induced immune responses, resulting in significant economic losses in the duck industry [8, 24]. However, few studies have been conducted on spleen injury induced by NDRV infection, particularly regarding the histological changes and gene expression patterns of the spleen. This study aimed to investigate target cells in the spleen following NDRV infection and identify DEGs under various pathological conditions via histopathology, immunohistochemistry analysis, and RNA-Seq technology. These findings hold significant reference value for elucidating the interaction mechanism between NDRV and its host.

ARV exhibits tropism for macrophages [17], and the target cells of MDRV infection include both macrophages and lymphocytes within the spleen. Similarly, in a single-cell resolution transcriptomic analysis of the bursa of Fabricius after infection with DRV, the proportions of dendritic cells (DCs) and macrophages significantly increased at 14 dpi with the HN10 strain of DRV. This finding underscores the critical role of DCs and macrophages in the long-term viral response [25]. Macrophages possess migratory properties that potentially contribute to the dissemination of viral infections [26]. This study provides evidence that splenic macrophages serve as the primary target cells for NDRV infection, thereby indicating a significant association between the formation of necrotic foci in the spleen and injury to macrophages. Macrophages play a pivotal role in host immunity against infection [27]. Pathogen invasion of macrophages not only facilitates the dissemination and persistence of infection by impairing phagocytosis and antigen presentation [28] but also triggers macrophage death through diverse mechanisms, thereby exacerbating tissue damage [29]. Subsequent RNA-Seq investigations revealed substantial enrichment of ferroptosis following NDRV infection. Macrophages are integral to the regulation of host iron metabolism. In 2017, it was initially reported that iron accumulation could induce ferroptosis in macrophages, and subsequent investigations demonstrated that Fer-1 could rescue liver damage caused by iron accumulation [30]. Within 2–4 h post fungal infection, macrophages exhibited a pronounced response in iron metabolism characterized by the upregulation of TfR1, DMT1, and ZIPs, along with the transcriptional downregulation of Fpn [31]. These findings suggest a potential association between macrophage iron metabolism, ferroptosis, and tissue damage.

Ferroptosis, an emerging form of regulated cell death, has been demonstrated to play a significant role in viral replication, transmission, and pathogenesis [32]. This includes its involvement in the infection and pathogenesis of avian influenza virus (AIV) [33], Newcastle disease virus (NDV) [34], infectious bronchitis virus (IBV), porcine reproductive and respiratory syndrome virus (PRRSV) [35], *Staphylococcus aureus* (*S. aureus*) [36], and other pathogens affecting livestock and poultry. IBV infection is a significant pathogenic factor in avian gout. Research has revealed that IBV infection induces disruptions in iron metabolism and glutathione (GSH) metabolism within the chicken kidney, resulting in the accumulation of lipid peroxides and subsequent host cell death. This pathological phenomenon may serve as a crucial mechanism underlying IBV-induced kidney injury in chickens. Furthermore, ferroptosis plays a prominent role in the pathogenesis of swine influenza virus (SIV). The aberrant expression of the iron-binding protein (IBP) following SIV infection disrupts intracellular iron metabolism, triggering host cell ferroptosis and facilitating viral proliferation, ultimately leading to severe acute respiratory disease [37].

The DEGs related to iron metabolism induced by NDRV infection were implicated in the transport, storage, and release of iron ions, indicating the crucial role of the iron metabolism pathway in NDRV-mediated regulation of host cell ferroptosis. As TfR1 is the main receptor protein that mediates iron entry into most cells, its expression has been the focus of many studies, and cells with high TfR1 expression are more sensitive to ferroptosis induced by erastin because of their intake of more iron ions [38]. A study of pathological myocardial hypertrophy revealed that circCmss upregulates TfR1 expression to induce ferroptosis in cardiomyocytes [39]. Nrf2, HSPB1, and other genes can regulate TfR1 expression and affect iron metabolism to modulate cellular sensitivity to ferroptosis [40, 41]. The induction of NDRV resulted in the upregulation of NCOA4 expression. NCOA4 facilitates the targeting of ferritin to lysosomes for autophagic degradation, leading to the release of iron ions, a process known as ferritinophagy [42]. An investigation of ferroptosis induced by NDV in tumor cells revealed that NDV triggers ferritin autophagy, leading to iron ion release and an enhanced Fenton reaction. Consequently, this promotes cellular ferroptosis and facilitates virus replication [34].

After viral infection, recognition of pathogen-associated molecular patterns (PAMPs) by the pattern recognition receptors (PRRs) of host cells initiates the signalling cascade of the innate immune response [43]. Toll-like receptors (TLRs) primarily mediate the recognition of RNA viruses [44]. This study revealed a significant increase in the expression levels of TLR2 and TLR5 following NDRV infection. Additionally, the NOD-like receptor and RIG-I-like receptor pathways have been implicated in the immune response of spleen cells after NDRV infection. Infection with ARV significantly upregulated the mRNA expression levels of TLR3 and TLR5 in host cells [45, 46]. MRV was found to induce innate immune responses in dendritic cells through RIG-I and MDA5 and activate the PI3K/Akt signalling pathway via clathrin-mediated endocytosis, leading to downstream EMSY phosphorylation and the regulation of ISG expression to inhibit virus proliferation, independent of the classical IFN-JAK-STAT signalling pathway [46]. NDRV infection activates the JAK-STAT, PI3K-Akt, NF-κB, and other signalling pathways. It is necessary to further clarify the interaction between NDRV and signal transduction proteins in these pathways and its impact on IFN-I signal transduction. When NDRV infection triggers the IFN signalling cascade, this process influences miRNA expression, subsequently affecting viral RNA expression. Certain miRNAs exert inhibitory effects on the translation of NDRV proteins. The interplay between viruses, IFN, and miRNAs represents a promising direction for future research [47]. The expression of IL-1β and IL-6 in Vero and DF-1 cells is upregulated during early ARV infection, promoting cell migration and infection spread through chemotactic response regulation [48]. While the production of inflammatory factors is crucial for the antiviral response, excessive expression can damage host cells [49], which may explain why NDRV activates IL-17 transcription and upregulates IL-1β and IL-8 transcription in the early stage of NDRV infection but is unable to effectively inhibit NDRV proliferation.

In summary, this study successfully elucidated the primary target cells and dynamic alterations in cell damage-associated genes following NDRV infection, revealing the pivotal role of ferroptosis in spleen damage. Moreover, these findings provide substantial data support for further investigations into cellular damage and host immune responses subsequent to avian orthoreovirus infection. Further exploration of the regulatory mechanism of NDRV in ferroptosis and its interplay with other modes of cell death in splenic necrosis will contribute to a comprehensive understanding of the pathogenic mechanisms underlying NDRV.

## Supplementary Information

**Additional file 1:** **Quantitative real-time PCR primers.**

**Additional file 2:** **Sequencing data of groups generated by an Illumina HiSeqTM 2000TM sequencer.**

**Additional file 3:** **Statistical results of sequencing data mapping to the reference genome.**

**Additional file 4:** **Significantly enriched KEGG pathways of the differentially expressed genes.**

## Data Availability

All transcriptome sequencing reads are openly available in the NCBI SRA, reference number PRJNA1174775.
