# Supplementary material for: Transcriptomic analysis revealed ferroptosis in ducklings with splenic necrosis induced by NDRV infection
Source: Vet Res. 2025 Mar 9;56:54. doi: 10.1186/s13567-025-01479-y (PMC11892222; doi:10.1186/s13567-025-01479-y)
Supplement: Supplementary file 1 — Additional file 1: Quantitative real-time PCR primers. [file 13567_2025_1479_MOESM1_ESM.docx]

**Additional file 1. Quantitative real-time PCR primers.**

| **Gene** | **Primer sequence (5’-3’)** | |
| --- | --- | --- |
|  | Forward primer | Reverse primer |
| STAT1 | ACTGCTGGAGCATTTGTTGC | ACAGCCGGAGTTCAGTCAAA |
| TLR5 | ACTGCTGGAGCATTTGTTGC | ACAGCCGGAGTTCAGTCAAA |
| IRF7 | AGGAGGTGGTGAATGACAGG | GACCTTGCACTTCTTCAGGC |
| IL1β | TCATCTTCTACCGCCTGGAC | GTAGGTGGCGATGTTGACCT |
| TfR1 | CTCCTTTGAGGCTGGTGAGG | CACTTGGTTCTTGGTGCTGC |
| Fpn | TTACCTTGGACATGCGCTGT | CCTCGAGTTCTTGTCCACCC |
| GPx4 | AAATGAGGAAAGACCGCGGT | CATGTCGAACTTCACCCCGT |
| PTGS2 | TCCACCGGTAGGACATGACT | GCCAGGCCCTTTCTTATGGT |
| β-actin | TGATATTGCTGCGCTCGTTG | AACCATCACACCCTGATGTCTG |
