# Supplementary material for: Transcriptomic analysis revealed ferroptosis in ducklings with splenic necrosis induced by NDRV infection
Source: Vet Res. 2025 Mar 9;56:54. doi: 10.1186/s13567-025-01479-y (PMC11892222; doi:10.1186/s13567-025-01479-y)
Supplement: Supplementary file 2 — Additional file 2: Sequencing data of groups generated by an Illumina HiSeqTM 2000TM sequencer. [file 13567_2025_1479_MOESM2_ESM.docx]

**Additional file 2. Sequencing data of groups generated by an Illumina HiSeqTM 2000TM sequencer.**

| Sample | Raw reads No. | Raw N (%) | Raw Q20 (%) | Raw Q30 (%) | Clean reads No. | Clean reads (%) |
| --- | --- | --- | --- | --- | --- | --- |
| Mock-1dpi | 51132436 | 0.002744 | 97.63 | 93.95 | 47587346 | 93.06 |
| Mock-2dpi | 43011046 | 0.000197 | 97.97 | 94.36 | 40496854 | 94.15 |
| Mock-3dpi | 47790280 | 0.002658 | 97.89 | 94.24 | 44915402 | 93.98 |
| Mock-5dpi | 42881582 | 0.000195 | 97.86 | 94.17 | 40240184 | 93.84 |
| Mock-7dpi | 45892314 | 0.000204 | 97.84 | 94.09 | 43055388 | 93.81 |
| NDRV-1dpi | 49969000 | 0.002841 | 98.07 | 94.62 | 45205578 | 94.42 |
| NDRV-2dpi | 44068654 | 0.000193 | 98.05 | 94.65 | 40629424 | 93.82 |
| NDRV-3dpi | 52777012 | 0.002659 | 97.88 | 94.21 | 46678594 | 93.91 |
| NDRV-5dpi | 43288612 | 0.000965 | 97.97 | 94.38 | 41328990 | 93.16 |
| NDRV-7dpi | 51547952 | 0.000217 | 97.77 | 93.95 | 43812138 | 93.6 |

Note: N (%) represents the percentage of fuzzy bases; Q30 (%) represents the numbers of bases with base recognition accuracy more than 99.9%; Q20 (%): represents the numbers of bases with base recognition accuracy more than 99%.
