# Supplementary material for: Transcriptomic analysis revealed ferroptosis in ducklings with splenic necrosis induced by NDRV infection
Source: Vet Res. 2025 Mar 9;56:54. doi: 10.1186/s13567-025-01479-y (PMC11892222; doi:10.1186/s13567-025-01479-y)
Supplement: Supplementary file 3 — Additional file 3: Statistical results of sequencing data mapping to the reference genome. [file 13567_2025_1479_MOESM3_ESM.docx]

**Additional file 3. Statistical results of sequencing data mapping to the reference genome.**

| Sample | Total Mapped | | Uniquely Mapped | | Multiple Mapped | | Mapped to Gene | | Mapped to Exon | |
| --- | --- | --- | --- | --- | --- | --- | --- | --- | --- | --- |
|  | Number | Rate (%) | Number | Rate (%) | Number | Rate (%) | Number | Rate (%) | Number | Rate (%) |
| Mock-1dpi | 41888291 | 88.02 | 38892876 | 92.85 | 2995415 | 7.15 | 37915416 | 97.49 | 34388119 | 90.7 |
| Mock-2dpi | 35869374 | 88.57 | 33394812 | 93.10 | 2474562 | 6.90 | 32607303 | 97.64 | 29879231 | 91.63 |
| Mock-3dpi | 39480918 | 87.90 | 36626618 | 92.77 | 2854300 | 7.23 | 35675172 | 97.4 | 31667617 | 88.77 |
| Mock-5dpi | 35156330 | 87.37 | 32220992 | 91.65 | 2935338 | 8.35 | 31379567 | 97.39 | 28303611 | 90.2 |
| Mock-7dpi | 38159304 | 88.63 | 34782147 | 91.15 | 3377157 | 8.85 | 33826999 | 97.25 | 30010755 | 88.72 |
| NDRV-1dpi | 41737390 | 88.79 | 38304087 | 91.77 | 3433303 | 8.23 | 37326163 | 97.45 | 33941726 | 90.93 |
| NDRV-2dpi | 36821000 | 88.73 | 33893167 | 92.05 | 2927833 | 7.95 | 33158375 | 97.83 | 31674306 | 95.52 |
| NDRV-3dpi | 43104807 | 88.14 | 38878093 | 90.19 | 4226714 | 9.81 | 37890907 | 97.46 | 34957510 | 92.26 |
| NDRV-5dpi | 35780661 | 88.14 | 33225390 | 92.86 | 2555271 | 7.14 | 32364133 | 97.41 | 29271138 | 90.44 |
| NDRV-7dpi | 42952749 | 89.02 | 36803590 | 85.68 | 6149159 | 14.32 | 35547379 | 96.59 | 32534310 | 91.52 |

Note: Total Mapped, the total number of sequences aligned to the reference genome, and its percentage is calculated as Total Mapped divided by Clean Reads. Uniquely Mapped, the total number of sequences mapped to only one position, and its percentage is calculated as Uniquely Mapped divided by total mapped. Multiple Mapped represents the total number of sequences aligned to multiple positions, with its percentage being Multiple Mapped divided by total Mapped. Mapped to Gene denotes the total number of reads aligned to gene regions, with its percentage being calculated as Mapped to gene divided by Uniquely Mapped. Mapped to Exon signifies the total number of reads aligned specifically to exon regions, and its percentage is calculated as Mapped to exon divided by Maaped to Gene.
