# Supplementary material for: Transcriptomic analysis revealed ferroptosis in ducklings with splenic necrosis induced by NDRV infection
Source: Vet Res. 2025 Mar 9;56:54. doi: 10.1186/s13567-025-01479-y (PMC11892222; doi:10.1186/s13567-025-01479-y)
Supplement: Supplementary file 4 — Additional file 4: Significantly enriched KEGG pathways of the differentially expressed genes. [file 13567_2025_1479_MOESM4_ESM.docx]

**Additional file 4. Significantly enriched KEGG pathways of the differentially expressed genes.**

| **Time** | **KEGG description** | **DEGs** | ***p*-value** |
| --- | --- | --- | --- |
| 1 dpi | ECM-receptor interaction | 9 | 3.23E-07 |
|  | Focal adhesion | 17 | 0.00022 |
|  | Antigen processing and presentation | 6 | 0.00493 |
|  | Phagosome | 11 | 0.00596 |
|  | PI3K-Akt signaling pathway | 21 | 0.00184 |
|  | Complement and coagulation cascades | 12 | 2.58E-06 |
|  | AGE-RAGE signaling pathway in diabetic complications | 9 | 0.00346 |
|  | Hematopoietic cell lineage | 6 | 0.03955 |
|  | Cytokine-cytokine receptor interaction | 12 | 0.03841 |
|  | Chemokine signaling pathway | 10 | 0.02801 |
|  | JAK-STAT signaling pathway | 1 | 0.00565 |
|  | NF-κB signaling pathway | 8 | 0.00593 |
|  | RIG-I-like receptor signaling pathway | 5 | 0.03540 |
|  | C-type lectin receptor signaling pathway | 12 | 0.04488 |
| 2 dpi | ECM-receptor interaction | 14 | 3.73E-06 |
|  | Complement and coagulation cascades | 15 | 9.06E-08 |
|  | AGE-RAGE signaling pathway in diabetic complications | 12 | 0.00034 |
|  | Chemokine signaling pathway | 15 | 0.00114 |
|  | Relaxin signaling pathway | 10 | 0.01855 |
|  | Cytokine-cytokine receptor interaction | 28 | 1.04E-08 |
|  | Antigen processing and presentation | 9 | 0.00011 |
|  | Primary immunodeficiency | 5 | 0.01357 |
|  | PI3K-Akt signaling pathway | 20 | 0.03330 |
|  | Phagosome | 24 | 1.03E-09 |
|  | Pathways in cancer | 39 | 2.03E-05 |
|  | NF-κB signaling pathway | 13 | 2.50E-05 |
|  | Toll-like receptor signaling pathway | 13 | 4.71E-05 |
|  | NOD-like receptor signaling pathway | 15 | 6.57E-05 |
|  | IL-17 signaling pathway | 7 | 0.02995 |
|  | Hematopoietic cell lineage | 8 | 0.01152 |
|  | RIG-I-like receptor signaling pathway | 9 | 0.00033 |
|  | C-type lectin receptor signaling pathway | 10 | 0.00650 |
|  | TNF signaling pathway | 13 | 0.00016 |
|  | Necroptosis | 13 | 0.00283 |
|  | JAK-STAT signaling pathway | 13 | 0.00365 |
|  | Apoptosis | 12 | 0.00608 |
|  | Apoptosis - multiple species | 4 | 0.03392 |
|  | Cytosolic DNA-sensing pathway | 6 | 0.00954 |
|  | HIF-1 signaling pathway | 10 | 0.00330 |
|  | Ferroptosis | 6 | 0.00541 |
|  | PPAR signaling pathway | 6 | 0.03448 |
| 3 dpi | Complement and coagulation cascades | 14 | 9.07E-06 |
|  | Cytokine-cytokine receptor interaction | 33 | 2.27E-09 |
|  | Phagosome | 27 | 9.83E-10 |
|  | Hematopoietic cell lineage | 11 | 0.00151 |
|  | NF-κB signaling pathway | 13 | 0.00025 |
|  | TNF signaling pathway | 12 | 0.00421 |
|  | Primary immunodeficiency | 5 | 0.03287 |
|  | Chemokine signaling pathway | 22 | 6.97E-06 |
|  | Ferroptosis | 6 | 0.01580 |
|  | Cytosolic DNA-sensing pathway | 8 | 0.00185 |
|  | Toll-like receptor signaling pathway | 17 | 1.57E-06 |
|  | B cell receptor signaling pathway | 9 | 0.00883 |
|  | JAK-STAT signaling pathway | 17 | 0.00056 |
|  | Antigen processing and presentation | 8 | 0.00280 |
|  | HIF-1 signaling pathway | 14 | 0.00016 |
|  | ECM-receptor interaction | 14 | 5.00E-05 |
|  | PPAR signaling pathway | 9 | 0.00319 |
|  | C-type lectin receptor signaling pathway | 13 | 0.00142 |
|  | NOD-like receptor signaling pathway | 16 | 0.00025 |
|  | AGE-RAGE signaling pathway in diabetic complications | 12 | 0.00251 |
|  | Necroptosis | 15 | 0.00310 |
|  | PI3K-Akt signaling pathway | 24 | 0.03390 |
| 5 dpi | IL-17 signaling pathway | 8 | 0.00401 |
|  | PPAR signaling pathway | 6 | 0.01780 |
|  | Complement and coagulation cascades | 9 | 0.00066 |
|  | TNF signaling pathway | 13 | 3.34E-05 |
|  | Hematopoietic cell lineage | 17 | 5.68E-10 |
|  | Cytokine-cytokine receptor interaction | 21 | 7.41E-06 |
|  | Focal adhesion | 25 | 1.14E-08 |
|  | Toll-like receptor signaling pathway | 11 | 0.00021 |
|  | Pathways in cancer | 32 | 0.00026 |
|  | Cell adhesion molecules (CAMs) | 11 | 0.00286 |
|  | JAK-STAT signaling pathway | 12 | 0.00288 |
|  | ECM-receptor interaction | 19 | 2.12E-17 |
|  | B cell receptor signaling pathway | 5 | 0.09668 |
|  | Intestinal immune network for IgA production | 6 | 0.00366 |
|  | PI3K-Akt signaling pathway | 26 | 3.76E-05 |
|  | Relaxin signaling pathway | 14 | 4.29E-05 |
|  | AGE-RAGE signaling pathway in diabetic complications | 12 | 8.07E-05 |
|  | Gap junction | 10 | 0.00062 |
|  | Platelet activation | 12 | 0.00093 |
|  | Phagosome | 13 | 0.00100 |
|  | NOD-like receptor signaling pathway | 10 | 0.00720 |
|  | Regulation of actin cytoskeleton | 14 | 0.00742 |
|  | NF-κB signaling pathway | 8 | 0.00797 |
|  | Chemokine signaling pathway | 11 | 0.01606 |
|  | Cytosolic DNA-sensing pathway | 5 | 0.02007 |
|  | Th17 cell differentiation | 8 | 0.02376 |
|  | Antigen processing and presentation | 5 | 0.02571 |
|  | Ferroptosis | 4 | 0.05000 |
| 7 dpi | Complement and coagulation cascades | 6 | 0.02691 |
|  | Cytosolic DNA-sensing pathway | 5 | 0.01588 |
|  | ECM-receptor interaction | 9 | 0.00125 |
|  | Gap junction | 10 | 0.00038 |
|  | Pathways in cancer | 25 | 0.01438 |
|  | Phagosome | 8 | 0.08405 |
|  | Platelet activation | 17 | 3.15E-07 |
|  | Relaxin signaling pathway | 12 | 0.00037 |
|  | TNF signaling pathway | 9 | 0.00492 |
|  | Chemokine signaling pathway | 13 | 0.00131 |
|  | Hematopoietic cell lineage | 12 | 5.64E-06 |
|  | AGE-RAGE signaling pathway in diabetic complications | 9 | 0.00325 |
|  | JAK-STAT signaling pathway | 11 | 0.00527 |
|  | PPAR signaling pathway | 6 | 0.01359 |
|  | NOD-like receptor signaling pathway | 9 | 0.01405 |
|  | Cytokine-cytokine receptor interaction | 13 | 0.01663 |
|  | cAMP signaling pathway | 12 | 0.01736 |
|  | Toll-like receptor signaling pathway | 7 | 0.02485 |
